# The Impact of a Pulmonary-Artery-Catheter-Based Protocol on Fluid and Catecholamine Administration in Early Sepsis

**DOI:** 10.1155/2012/161879

**Published:** 2012-02-21

**Authors:** Carina Bethlehem, Frouwke M. Groenwold, Hanneke Buter, W. Peter Kingma, Michael A. Kuiper, Fellery de Lange, Paul Elbers, Henk Groen, Eric N. van Roon, E. Christiaan Boerma

**Affiliations:** ^1^Department of Intensive Care, Medical Centre Leeuwarden, P.O. Box 888, 8901 BR Leeuwarden, The Netherlands; ^2^Department of Cardiothoracic Anaesthesiology, Medical Centre Leeuwarden, P.O. Box 888, 8901 BR Leeuwarden, The Netherlands; ^3^Department of Epidemiology, University Medical Centre Groningen, P.O. Box 30001, 9700 RB Groningen, The Netherlands; ^4^Department of Clinical Pharmacy and Clinical Pharmacology, Medical Centre Leeuwarden, P.O. Box 888, 8901 BR Leeuwarden, The Netherlands

## Abstract

*Objective*. The pulmonary artery catheter (PAC) remains topic of debate. Despite abundant data, it is of note that many trials did not incorporate a treatment protocol. *Methods*. We retrospectively evaluated fluid balances and catecholamine doses in septic patients after the introduction of a PAC-based treatment protocol in comparison to historic controls. *Results*. 2 × 70 patients were included. The first day the PAC group had a significantly higher positive fluid balance in comparison to controls (6.1 ± 2.6 versus 3.8 ± 2.4 litre, *P* < 0.001). After 7 days the cumulative fluid balance in the PAC group was significantly lower than in controls (9.4 ± 7.4 versus 13 ± 7.6 litre, *P* = 0.001). Maximum dose of norepinephrine was significantly higher in the PAC group. Compared to controls this was associated with a significant reduction in ventilator and ICU days. *Conclusions*. Introduction of a PAC-based treatment protocol in sepsis changed the administration of fluid and vasopressors significantly.

## 1. Introduction

The pulmonary artery catheter (PAC) by Swan and Ganz, in the setting of critically ill patients, was originally introduced to “apply physiologic principles to the understanding of the circulatory abnormalities characterizing an illness in an individual patient, and to provide a rational basis for selection of therapy with objective, quantitative assessment of patient response” [1, 2]. In the following decades, this mechanistic perspective on the clinical relevance of PAC and other monitoring devices was gradually abandoned and replaced by “evidence-based medicine,” with emphasis on its potential value to reduce morbidity and mortality. Ever since, multiple randomised controlled trials in different subsets of ICU patients have been performed, to evaluate the use of PAC to improve outcome [3–7]. Lack of consistency in the results of these trials have led many to believe that the use of PAC should be done with great restraint [8]. Others, however, have stressed the potential methodological drawbacks of these trials, that may obscure underlying beneficial effects of the use of PAC; correct measurement, correct interpretation, and correct application of PAC-derived data are all essential to the final result [9, 10]. Today, many aspects of such methodological flaws have been acknowledged. Errors in measurements [11, 12], delay in insertion of PAC in acutely ill patients [13], misinterpretation of static filling pressures as a marker of preload [14], absence of therapeutic strategies [6, 7], as well as faulty supranormal endpoints [15] have all been reported. Furthermore, over the years the use of PAC has shifted from intermittently measuring static filling pressures towards a continuous indicator of (dis)balance between oxygen supply (cardiac output) and consumption (mixed venous oxygen saturation, SvO_2_). Furthermore it has now become a tool for the assessment of functional hemodynamic parameters, such as fluid responsiveness. To our knowledge, data on the effect of a PAC-based protocol, that integrates most of these aspects seem to be lacking.

In the present study we aimed to evaluate the influence of a PAC-based protocol on fluid administration and catecholamine use of well-trained intensivists in the specific setting of critically ill patients with early-phase severe sepsis/septic shock. We chose this particular group of patients, under the assumption that (a change in) hemodynamic management might have considerable potential impact on patient morbidity. Primary endpoints were the fluid balance after 24 hours and 7 days and maximum dose of dopamine and norepinephrine within the first 24 hours. Secondary outcome variables were days on the ventilator and length of stay (LOS) ICU.

## 2. Material and Methods

### 2.1. Patients

The study was performed in a closed-format 22-bed mixed ICU in a tertiary teaching hospital. After the introduction of a PAC-based protocol for hemodynamic management as standard treatment for patients with sepsis as primary reason for ICU admittance, all patients ≥18 years with severe sepsis and septic shock, according to international criteria [16], were included in the study during an 18-month period in 2007-2008. The historic control group was recruited from our database in a 2-year period in 2005-2006 and matched for sepsis criteria in a 1 : 1 ratio from a consecutive period prior to implementation of the protocol. The experiment was conducted with the understanding and the consent of the human subject. According to applicable laws the need for ethical approval or individual consent was waived.

### 2.2. Protocol

**Figure 1:**
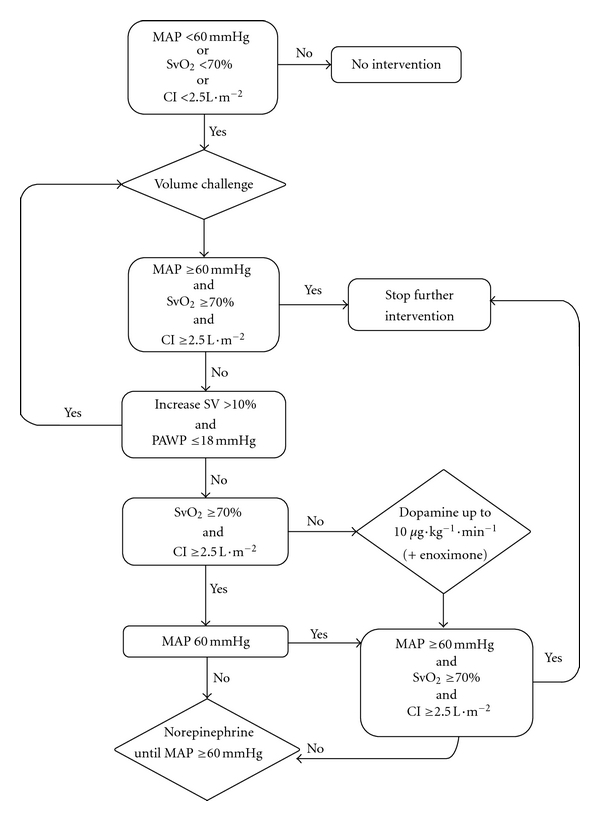
Treatment algorithm.

 During the study period hemodynamic assessment in patients with severe sepsis or septic shock was achieved through continuous invasive monitoring of arterial blood pressure and right heart catheterisation with continuous cardiac output and SvO_2_ measurement (Vigilance, Edwards Lifesciences, Saint-Prex, Switzerland) within 4 hours after ICU admittance. Until a PAC was in place, the use of fluids and vasoactive drugs was at the discretion of the attending physician, aiming at a minimal mean arterial pressure (MAP) of 60 mmHg. After insertion and calibration of the PAC, treatment of circulatory failure was aimed at a MAP ≥60 mmHg in combination with a cardiac index (CI) ≥2.5 L/m^2^/min and an SVO_2_ ≥70%. Achievement of these endpoints was performed in the following strict hierarchical order. (1) Exclusion of fluid responsiveness by repeated infusions of at least 250 mL crystalloids, colloids, or blood products, until the increase in left ventricular stroke volume was less than 10% or until the pulmonary artery wedge pressure exceeded 18 mmHg. Fluid administration was also stopped in case hemodynamic endpoints were fulfilled. (2) Treatment of inadequate systemic oxygen supply, defined as a cardiac index <2.5 L/m^2^/min or central venous oxygen saturation <70%, with dopamine administered at up to 10 *μ*g/kg/min and additional enoximone in the event of an inadequate response to dopamine. (3) Reversal of hypotension with norepinephrine in case of MAP <60 mmHg despite the aforementioned steps (Figure 1). Feedback on adequacy of PAC measurements and compliance with the protocol was given to the attending intensivists on a daily basis by an independent observer.

In the control group hemodynamic monitoring consisted of an indwelling arterial catheter and central venous line. Treatment was aimed at a MAP ≥60 mmHg and central venous pressure (CVP) between 8 and 12 mmHg. A closed-format setting, as well as protocols for the use of antibiotics (including selective decontamination of the digestive tract), tight glucose regulation, low tidal volume ventilation, weaning, (enteral) nutrition, activated protein C and steroid administration, and a general red blood cell transfusion trigger (hematocrit <25%) were unaltered during the study and control period.

### 2.3. Data Collection

The following data were recorded at baseline: demographic characteristics; severity of illness and predicted mortality consistent with APACHE IV [17], SOFA [18] (calculated over the first 24 hours following ICU admission), and RIFLE [19] scores; hemodynamic data including fluid balance and dose of vasoactive drugs; results of standard laboratory tests, including blood gases, arterial lactate concentrations, blood cultures, and cultures of specimens sampled from each presumed site of infection. Daily routine recordings consisted of hemodynamic data, fluid balance, dose of vasoactive drugs, arterial lactate concentrations, and blood gases; SOFA and RIFLE scores were calculated daily during each patient's ICU stay. The presence of ARDS was retrospectively established by an independent observer by chest X-ray assessment in combination with gas exchange criteria [20]. Survival status was confirmed for each subject at the end of their hospitalisation.

### 2.4. Statistical Analysis

For continuous variables, data are presented as mean ± SD or as medians and interquartile ranges (IQRs) in case of nonnormal distribution. Differences in baseline values and outcome parameters between groups were compared using an independent sample *t*-test, or Mann-Whitney test in case of nonnormal distribution. Comparison of mortality rates across different treatment strategies was performed using the *χ* ^2^ test. A two-sided *P* value of <0.05 was considered statistically significant. Confounding of the group effect on the primary endpoint was analysed using multiple regression analyses. Variables with significant group differences at baseline were entered individually and in combination in the regression model to detect significant confounding effects. The Statistical Package for Social Sciences (SPSS 15.1 for Windows, Chicago, IL, USA) was used for statistical analyses. Sample size was based on the following assumptions. According to a random sample of 20 protocol-treated patients we estimated the fluid balance after 24 hours 5.7 ± 2.5 litres. With an alpha of 0.05 and a power of 0.9, it would require a sample size of 2 × 66 patients to detect a difference of at least 1 litre.

## 3. Results

In 2007 and 2008 70 patients fulfilling the criteria for severe sepsis or septic shock were included in the study; 70 matched control patients were recruited from 2004 to 2006. Protocolized resuscitation and pulmonary artery catheterisation was performed successfully in all patients within 4 hours of ICU admittance. No PAC-related complications, including pneumothorax, line-related sepsis, or knotting were reported; median duration of PA catheterisation was 4 days. Baseline characteristics were balanced between groups with the exception of a significantly higher age, lactate, and RIFLE score in the control group and higher SOFA score in the PAC group (Table 1).

**Figure 2:**
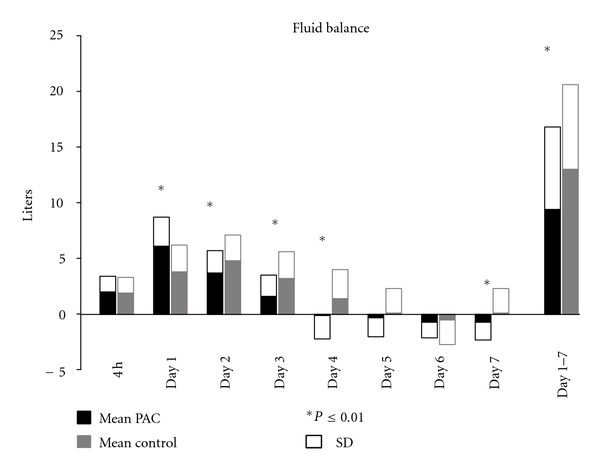
Fluid balances in the first week.

*Primary Outcome*. During the first 24 hours patients in the PAC group had a significantly higher positive fluid balance in comparison to controls (6.1 ± 2.6 versus 3.8 ± 2.4 litre, *P* < 0.001). However, after 7 days the cumulative fluid balance in the PAC group was significantly lower than in controls (9.4 ± 7.4 versus  13 ± 7.6  litre, *P* = 0.001; Table 2, Figure 2). Use of norepinephrine was significantly higher in the PAC group, both in dose and number of patients, but no difference in the use of dopamine between groups was observed (Table 2). Multiple linear regression analyses showed that the statistically significant difference in fluid balance after day 1 between the groups was not altered after correction for age, RIFLE score, lactate an SOFA score (*P* < 0.001).

**Figure 3:**
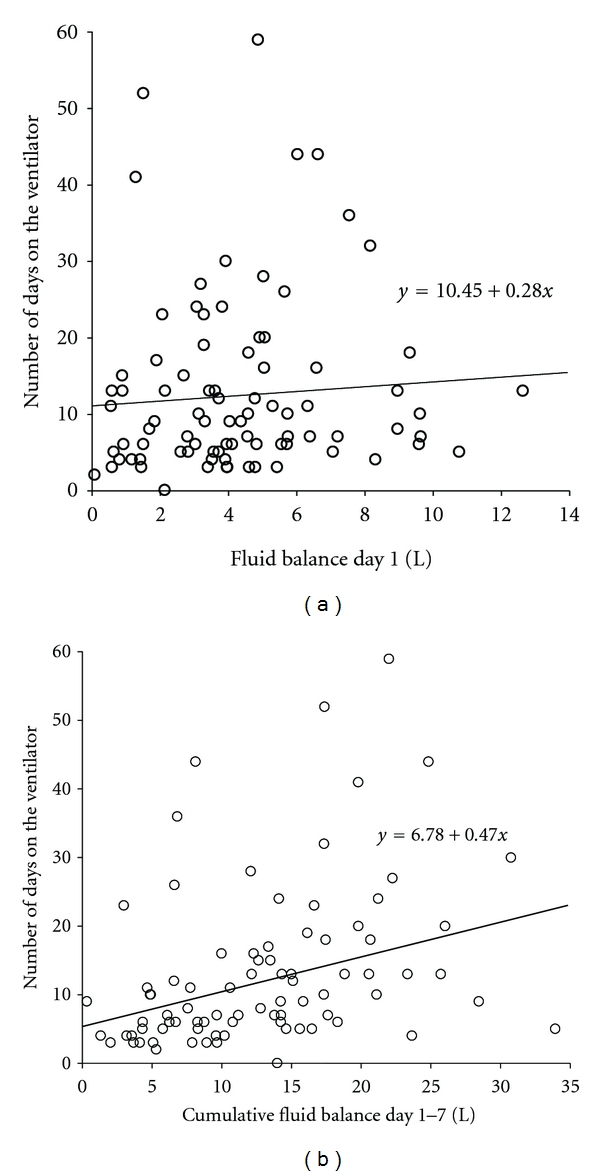
Linear regression analysis on the relation between the total number of days on the ventilator and the fluid balance after 24 hours (a), as well as the fluid balance after 7 days (b).

*Secondary Outcome*. Median number of days on the ventilator was significantly lower in the PAC group in comparison to controls: 7 (5–11) versus 10 (6–18) days, *P* = 0.01 (Table 3). This was accompanied by a significantly shorter LOS ICU for patients in the PAC group, as compared to controls: 9 (6–13) versus 14 (7–28) days, *P* < 0.001. Post hoc univariate analysis revealed a significant correlation between the cumulative fluid balance after 7 days and both number of days on the ventilator (*r*_*s*_ = 0.47, *P* < 0.001) as well as LOS ICU (*r*_*s*_ = 0.43, *P* < 0.001). However, there was no correlation between the fluid balance on day 1 and number of days on the ventilator or LOS ICU (*r*_*s*_ = 0.17, *P* = 0.06, and *r*_*s*_ = 0.13, *P* = 0.12, resp.; Figure 3).

## 4. Discussion

In the present study implementation of a PAC-based protocol for the hemodynamic management of patients with severe sepsis and septic shock was associated with a considerable impact on the use of volume resuscitation and vasopressors, both in timing and total volume. In comparison to historic controls, the PAC group received significantly more fluids during the first 24 hours. Interestingly this was accompanied by a significant reduction in total fluid administration in the first 7 days. These differences were not only statistically significant, but also associated with clinically relevant endpoints: reduction of days on the ventilator and LOS ICU.

In this respect, the setting in which the PAC-based protocol was tested seems to be of great importance. We specifically selected patients with assumed perfusion abnormalities. In this setting of patients with severe sepsis or septic shock, we anticipated a high likelihood to detect differences in the early management of fluids and vasoactive drugs between conventional and PAC-based hemodynamic treatment. This is in contrast to other groups of patients, in which hemodynamic management may not be of equal importance, for example, in routine noncardiac surgery [21].

Despite an overwhelming number of trials, addressing the use of PAC in different clinical subsets, there are not many data specifically focussed on (differences) in the use of fluids, inotropes, and vasopressors, as a result of a PAC-based treatment algorithm. The vast majority of studies did not incorporate hemodynamic endpoints and/or treatment protocols and failed to report how the use of PAC changed therapeutic behaviour [7, 22]. Other studies aimed for supranormal endpoints (CI, SvO_2_) generally considered faulty in hindsight; interestingly in these trials only a minority of patients fulfilled endpoints [3, 15]. Furthermore, many protocols were based on the incorrect assumption that static filling pressures could predict cardiac response to volume infusion [14] or formation of pulmonary oedema [23].

The presented dynamics in fluid administration (more fluids in the early phase, less fluids later) show similarities with previous data from hemodynamic endpoint-driven treatment protocols in sepsis. After a S_(c)_vO_2_ -based protocol differences in the use of fluids in comparison to conventional treatment were observed after 6 hours, rather than after 3 days [24]. In a retrospective study in patients with septic shock and ARDS, both initial fluid frontloading and subsequent fluid restriction were identified as markers of morbidity and mortality [25]. In accordance with our data, this was associated with increased use of norepinephrine, but not dopamine. The importance of timing was illustrated by the fact that the use of PAC in ARDS, after a mean period of admittance to the ICU of >40 hours and a fluid intake >4900 mL, was not associated with improvement in outcome [13]. Interestingly, we observed an association between LOS ICU/number of days on the ventilator and the cumulative fluid balance after 7 days, but not after 24 hours. This may be explained by the fact that pulmonary oedema formation in sepsis may not occur during (early) volume loading in the steep part of the cardiac function curve, as opposed to (late) volume loading in the horizontal part of the curve [26].

It seems unlikely that the observed changes in fluid dynamics and vasopressor administration are restricted to the use of the PAC itself. Guidance by other physiologic variables, derived from pulse contour analysis or oesophageal Doppler monitoring, were also associated with a change in therapeutic behaviour [27–29].

Several limitations of the study need to be addressed. Comparison between an intervention group and historic controls may be biased by unknown changes in patient management over time. The imbalance in lactate at baseline between groups might reflect significant differences in level of resuscitation or case mix and, therefore, create a bias in interpretation of the fluid balances. To “correct” this to some extent we performed a multiple linear regression analysis that included the difference in baseline lactate between groups, and its potential influence in fluid balance after day 1. After correction for age, RIFLE score, lactate, and SOFA score, the impact of the protocol remained highly significant for the primary endpoints (*P* < 0.001). Similar considerations need to be taken into account with respect to the imbalance in RIFLE score at baseline. The presence of acute renal failure at baseline is likely to be associated with LOS ICU and mortality. Alternatively, a positive fluid balance itself has also been identified as an independent risk factor for the occurrence of acute renal failure [30].

A single centre setup determines both skills in insertion of PAC and correct measurements, as well as the use of fluids and vasoactive drugs “according to the discretion of the attending physician.” Extrapolation to other settings should therefore be done with great restraint. Although the relation between primary and secondary outcome variables appear to be relevant, one should realize that the number of days on the ventilator and LOS ICU is surrogate endpoint for ICU treatment. However, the number of patients in this study was not adequate to detect potential differences in hospital mortality. At best, our results can be considered as hypothesis generating, rather than conclusive. Nevertheless, the data seem to indicate that a PAC-based treatment protocol, applied to a very early phase of a disease state with a high a priori chance on hemodynamic-related morbidity and mortality, has considerable impact on fluid and vasopressor management in comparison to nonprotocolized treatment, both in absolute numbers and dynamics. Future studies with adequate design are necessary to establish its potential for mortality reduction.

## Figures and Tables

**Table 1:** Baseline characteristics.

Variables	PAC (*n* = 70)	Control (*n* = 70)	*P* value
Male, *n* (%)	42 (61)	39 (56)	0.49
Age	62 ± 16	67 ± 13	0.02
APACHE IV	90 ± 47	88 ± 29	0.73
Predicted mortality APACHE IV (%)	43 ± 21	39 ± 16	0.36
SOFA	10 ± 3	8 ± 3	0.03
Source of infection			
Lung	19	16	
Abdominal	34	37	
Urinary tract	4	5	
Other	13	12	
ARDS (*n*)	4	5	0.19
Mean arterial pressure, mmHg	71 ± 12	68 ± 15	0.18
Heart rate, beats/min	110 ± 17	109 ± 20	0.82
Central venous pressure, mmHg	13 ± 5	12 ± 5	0.84
Ventilator, use of, *n* (%)	69 (99)	69 (99)	1.00
PEEP, cm H_2_O	13 ± 3	13 ± 3	0.88
Lactate, mmol/L	2.4 (1.4–4.3)	3.5 (2.7–5.4)	0.001
RIFLE score on admission	0 (0-1)	0 (0–2)	0.002

PAC: pulmonary artery catheter, APACHE: acute physiology and chronic health evaluation, SOFA: sequential organ failure assessment, PEEP: positive end expiratory pressure, RIFLE: risk injury failure loss and endstage. Data are presented as mean ± SD, median (IQR) or as numbers (%).

**Table 2:** Primary outcome variables: fluid balance and use of vasoactive drugs.

	PAC (*n* = 70)	Control (*n* = 70)	*P* value
Fluid balance day 0–4 hours (L)	2.0 ± 1.4	1.9 ± 1.4	0.79
Fluid balance day 1	6.1 ± 2.6	3.8 ± 2.4	0.000
Fluid balance day 2	3.7 ± 2.0	4.8 ± 2.3	0.002
Fluid balance day 3	1.6 ± 1.9	3.2 ± 2.4	0.000
Fluid balance day 4	−0.1 ± 2.1	1.4 ± 2.6	0.000
Fluid balance day 5	−0.3 ± 1.7	0.1 ± 2.2	0.13
Fluid balance day 6	−0.7 ± 1.4	−0.5 ± 2.1	0.26
Fluid balance day 7	−0.7 ± 1.6	0.1 ± 2.2	0.01
Fluid balance day 1–7	9.4 ± 7.4	13 ± 7.6	0.002
Maximum dose norepinephrine (*μ*g/kg/min, *n*)	0.12 (0.03–0.19), 59	0.02 (0–0.17), 39	0.000
Maximum dose dopamine (*μ*g/kg/min, *n*)	7.02 (4.7–9.8), 65	7.7 (4.7–9.6), 66	0.79

PAC: pulmonary artery catheter. Data are presented as mean ± SD, median (IQR) or as numbers.

**Table 3:** Secondary outcome variables: morbidity and mortality.

Variables	PAC (*n* = 70)	Control (*n* = 70)	*P* value
Days on ventilator	7 (5–11)	10 (6–18)	0.01
PO_2_/FiO_2_ ratio, worst (mmHg)	196 ± 81	158 ± 64	0.003
CVVH, *n *	28	35	0.24
CVVH, days	0 (−5)	0 (0–8)	0.08
RIFLE score, highest	2 (0–3)	3 (1–3)	0.02
LOS ICU (days)	9 (6–13)	14 (7–28)	0.001
LOS hospital	24 (14–40)	30 (17–51)	0.16
Cumulative SOFA score day 1–5	39 ± 15	40 ± 16	0.67
Cumulative SOFA score day 1–5 survivors	39 ± 12	38 ± 16	0.63
Mortality ICU (*n*, %)	15 (21)	21 (30)	0.33
Mortality hospital (%)	17 (24)	27 (39)	0.10

PAC: pulmonary artery catheter, FiO_2_: inspiratory oxygen fraction, CVVH: continous veno venous hemofiltration, RIFLE: risk injury failure loss and endstage, LOS: length of stay, SOFA: sequential organ failure assessment. Data are presented as mean ± SD, median (IQR) or as numbers (%).

## References

[1] Swan HJC, Ganz W (1975). Use of balloon flotation catheters in critically ill patients. *Surgical Clinics of North America*.

[2] Swan HJ, Ganz W, Forrester J, Marcus H, Diamond G, Chonette D (1970). Catheterization of the heart in man with use of a flow-directed balloon-tipped catheter. *New England Journal of Medicine*.

[3] Sandham JD, Hull RD, Frederick Brant R (2003). A randomized, controlled trial of the use of pulmonary-artery catheters in high-risk surgical patients. *New England Journal of Medicine*.

[4] Bender JS, Smith-Meek MA, Jones CE (1997). Routine pulmonary artery catheterization does not reduce morbidity and mortality of elective vascular surgery: results of a prospective, randomized trial. *Annals of Surgery*.

[5] Rhodes A, Cusack RJ, Newman PJ, Grounds MR, Bennett DE (2002). A randomised, controlled trial of the pulmonary artery catheter in critically ill patients. *Intensive Care Medicine*.

[6] Richard C, Warszawski J, Anguel N (2003). Early use of the pulmonary artery catheter and outcomes in patients with shock and acute respiratory distress syndrome: a randomized controlled trial. *Journal of the American Medical Association*.

[7] Harvey S, Harrison DA, Singer M (2005). Assessment of the clinical effectiveness of pulmonary artery catheters in management of patients in intensive care (PAC-Man): a randomised controlled trial. *The Lancet*.

[8] Reade MC, Angus DC (2006). PAC-Man: game over for the pulmonary artery catheter?. *Critical Care*.

[9] Vincent JL, Pinsky MR, Sprung CL (2008). The pulmonary artery catheter: in medio virtus. *Critical Care Medicine*.

[10] Brandstetter RD, Grant GR, Estilo M, Rahim F, Singh K, Gitler B (1998). Swan-Ganz catheter: misconceptions, pitfalls, and incomplete user knowledge—an identified trilogy in need of correction. *Heart and Lung*.

[11] Morris AH, Chapman RH, Gardner RM (1985). Frequency of wedge pressure errors in the ICU. *Critical Care Medicine*.

[12] Gnaegi A, Feihl F, Perret C (1997). Intensive care physicians’ insufficient knowledge of right-heart catheterization at the bedside: time to act?. *Critical Care Medicine*.

[13] Wheeler AP, Bernard GR, Thompson BT (2006). Pulmonary-artery versus central venous catheter to guide treatment of acute lung injury. *New England Journal of Medicine*.

[14] Kumar A, Anel R, Bunnell E (2004). Pulmonary artery occlusion pressure and central venous pressure fail to predict ventricular filling volume, cardiac performance, or the response to volume infusion in normal subjects. *Critical Care Medicine*.

[15] Gattinoni L, Brazzi L, Pelosi P (1995). A trial of goal-oriented hemodynamic therapy in critically ill patients. *New England Journal of Medicine*.

[16] Levy MM, Fink MP, Marshall JC (2003). 2001 SCCM/ESICM/ACCP/ATS/SIS International Sepsis Definitions Conference. *Intensive Care Medicine*.

[17] Zimmerman JE, Kramer AA, McNair DS, Malila FM (2006). Acute Physiology and Chronic Health Evaluation (APACHE) IV: hospital mortality assessment for today’s critically ill patients. *Critical Care Medicine*.

[18] Vincent J-L, De Mendonça A, Cantraine F (1998). Use of the SOFA score to assess the incidence of organ dysfunction/failure in intensive care units: results of a multicenter, prospective study. *Critical Care Medicine*.

[19] Bellomo R, Ronco C, Kellum JA, Mehta RL, Palevsky P (2004). Acute renal failure—definition, outcome measures, animal models, fluid therapy and information technology needs: the Second International Consensus Conference of the Acute Dialysis Quality Initiative (ADQI) Group. *Critical Care*.

[20] Bernard GR, Artigas A, Brigham KL (1994). Report of the American-European consensus conference on ARDS: definitions, mechanisms, relevant outcomes and clinical trial coordination. *Intensive Care Medicine*.

[21] Schultz RJ, Whitfield GF, Lamura JJ (1985). The role of physiologic monitoring in patients with fractures of the hip. *Journal of Trauma*.

[22] Shah MR, Hasselblad V, Stevenson LW (2005). Impact of the pulmonary artery catheter in critically ill patients: meta-analysis of randomized clinical trials. *Journal of the American Medical Association*.

[23] Eisenberg PR, Hansbrough JR, Anderson D, Schuster DP (1987). A prospective study of lung water measurements during patient management in an intensive care unit. *American Review of Respiratory Disease*.

[24] Rivers E, Nguyen B, Havstad S (2001). Early goal-directed therapy in the treatment of severe sepsis and septic shock. *New England Journal of Medicine*.

[25] Murphy CV, Schramm GE, Doherty JA (2009). The importance of fluid management in acute lung injury secondary to septic shock. *Chest*.

[26] Van Der Heijden M, Verheij J, Van Nieuw Amerongen GP, Groeneveld ABJ (2009). Crystalloid or colloid fluid loading and pulmonary permeability, edema, and injury in septic and nonseptic critically ill patients with hypovolemia. *Critical Care Medicine*.

[27] Goepfert MSG, Reuter DA, Akyol D, Lamm P, Kilger E, Goetz AE (2007). Goal-directed fluid management reduces vasopressor and catecholamine use in cardiac surgery patients. *Intensive Care Medicine*.

[28] Lopes MR, Oliveira MA, Pereira VOS, Lemos IPB, Auler JOC, Michard F (2007). Goal-directed fluid management based on pulse pressure variation monitoring during high-risk surgery: a pilot randomized controlled trial. *Critical Care*.

[29] Wakeling HG, McFall MR, Jenkins CS (2005). Intraoperative oesophageal Doppler guided fluid management shortens postoperative hospital stay after major bowel surgery. *British Journal of Anaesthesia*.

[30] Payen D, de Pont AC, Sakr Y, Spies C, Reinhart K, Vincent JL (2008). A positive fluid balance is associated with a worse outcome in patients with acute renal failure. *Critical Care*.

